# The circadian system modulates the cortisol awakening response in humans

**DOI:** 10.3389/fnins.2022.995452

**Published:** 2022-11-03

**Authors:** Nicole P. Bowles, Saurabh S. Thosar, Matthew P. Butler, Noal A. Clemons, LaTroy D. Robinson, Omar H. Ordaz, Maya X. Herzig, Andrew W. McHill, Sean P. M. Rice, Jonathan Emens, Steven A. Shea

**Affiliations:** ^1^Oregon Institute of Occupational Health Sciences, Oregon Health and Science University, Portland, OR, United States; ^2^Knight Cardiovascular Institute, School of Medicine, Oregon Health and Science University, Portland, OR, United States; ^3^School of Nursing, Oregon Health and Science University, Portland, OR, United States; ^4^OHSU-PSU School of Public Health, Oregon Health and Science University, Portland, OR, United States; ^5^Department of Behavioral Neuroscience, Oregon Health and Science University, Portland, OR, United States; ^6^Department of Psychiatry, Oregon Health and Science University, Portland, OR, United States; ^7^VA Portland Health Care System, Portland, OR, United States

**Keywords:** HPA-axis, chronic stress, time of day, ACTH, sleep, circadian, cortisol, awakening

## Abstract

**Background:**

In humans, circulating cortisol usually peaks 30–60 min after awakening from nocturnal sleep, this is commonly referred to as the cortisol awakening response (CAR). We examined the extent to which the CAR is influenced by the circadian system, independent of behaviors including sleep.

**Materials and methods:**

We examined the CAR in 34 adults (20 female) using two complementary multiday in-laboratory circadian protocols performed in dim light, throughout which behavioral factors were uniformly distributed across the 24-hour circadian cycle. Protocol 1 consisted of 10 identical consecutive 5-hour 20-minute sleep/wake cycles, and protocol 2 consisted of 5 identical consecutive 18-hour sleep/wake cycles. Salivary melatonin was used as the circadian phase marker (0° = dim light melatonin onset). During each sleep/wake cycle, salivary cortisol was measured upon scheduled awakening and 50-minutes later, with the change in cortisol defined as the CAR. Cosinor analyses were used to detect any significant circadian rhythmicity in the CAR. In secondary analyses, we adjusted the models for time awake before lights on, total sleep time, percent of rapid eye movement (REM) sleep, and percent of non-rapid eye movement (NREM) sleep.

**Results:**

Both protocols revealed a similar circadian rhythm in the CAR, with peaks occurring at a circadian phase corresponding to 3:40–3:45 a.m., with no detectable CAR during the circadian phases corresponding to the afternoon. In addition to the sinusoidal component of the circadian rhythm, total sleep time was also associated with the CAR for protocol 1. The percent of sleep spent in REM or NREM sleep were not associated with the CAR in either protocol.

**Conclusion:**

Our results show that the CAR exhibits a robust circadian rhythm that persists even after adjusting for prior sleep. Presuming that the CAR optimizes physiological responses to the anticipated stressors related to awakening, these findings may have implications for shift workers who wake up at unusual circadian phases. A blunted CAR in shift workers upon awakening in the evening may result in diminished responses to stressors.

## Introduction

Shortly after awakening from nocturnal sleep, initial cortisol levels increase by 50% or more, a phenomenon known as the cortisol awakening response (CAR) (Pruessner et al., 1997; Clow et al., 2004). This CAR and associated physiological activation are argued to help prepare the body for the anticipated stressors of the day after the nocturnal rest period (Schlotz et al., 2004; Fries et al., 2009; Law et al., 2013). These changes include preparation for altered posture, increased energy demands, and social interactions (Wetherell et al., 2015; Ogasawara et al., 2022). Although the CAR may be considered a physiological adaptation in healthy humans, the traditionally assessed CAR after nocturnal sleep coincides with the period of the day with the greatest risk for serious adverse cardiovascular events (Muller et al., 1985; Manfredini et al., 2005; Thosar et al., 2018a) and it is conceivable that the CAR could be involved in such events in vulnerable individuals. Several studies show that the CAR is associated with prior-day experiences and anticipation of stress for the day ahead (Adam et al., 2006; Fries et al., 2009), but the neurobiological mechanisms that generate the CAR have not been fully elucidated. While the CAR requires awakening from sleep (Vargas and Lopez-Duran, 2020) or the anticipation of awakening (Born et al., 1999), a CAR can be observed following daytime sleep, including naps with the magnitude of the CAR varying according to the timing of the sleep episode (Späth-Schwalbe et al., 1992; Griefahn and Robens, 2008; Devine and Wolf, 2016). These daily variations in the CAR in response to awakening from sleep suggest that CAR dynamics could be regulated by internal circadian mechanisms as well as responses to daily behaviors (awakening) or environmental cues (i.e., light).

In clinical populations, the CAR has been considered a marker for depression among other pathologies (Vrshek-Schallhorn et al., 2013). However, inconsistencies between studies (e.g., an attenuated vs. increased CAR in association with the level of depression (Dedovic and Ngiam, 2015)), prevent the use of the CAR as a standard diagnostic tool. In recent years consensus guidelines for CAR assessment have been developed as an effort to reduce the variabilities both between and within study measurements (Stalder et al., 2016). The consensus guidelines note that the time of awakening is likely to impact CAR levels and CAR measurements should be postponed if the participant recently experienced circadian disruption (e.g., shift work or travel across time zones). While, for example, the collection of CAR following consecutive morning and night shifts intimates that differences in the CAR when measured after night-sleep compared to day-sleep would be at least partly caused by measurements occurring at different circadian phases (Griefahn and Robens, 2010), this and similar studies are not designed to fully separate the contributions of the endogenous circadian system and behaviors such as sleep. Thus, it remains unclear to what extent the CAR is dependent on the endogenous circadian system. Based on these previous findings, we hypothesized that the endogenous circadian system modulates the CAR. Findings in agreement with our hypothesis would suggest that measurement of an individual’s internal circadian clock time with respect to the time of CAR assessment would further improve consistency of the measure. Finally, if the circadian system modulates the CAR, then the CAR is likely to be perturbed in shift work when people wake up at unusual circadian phases. This perturbation could lead to sub-optimal physiological responses to stressors in shift-workers, and this could conceivably be related to the increased risk of cardiovascular disease that accompanies shift work (Torquati et al., 2018). Therefore, understanding the circadian mechanisms that influence the CAR is potentially important for certain diagnoses, as well as the adverse health effects of night shift work.

Our aim was to determine, by using the gold standard approach for assessing intrinsic circadian rhythms in humans, whether the endogenous circadian system modulates the magnitude of the CAR when awakenings occur at different circadian phases. We demonstrate the robustness of the circadian rhythm of the CAR using two circadian protocols, with results that can be generalized across sexes and ages based on the different populations studied in the two protocols.

## Materials and methods

**FIGURE 1:**
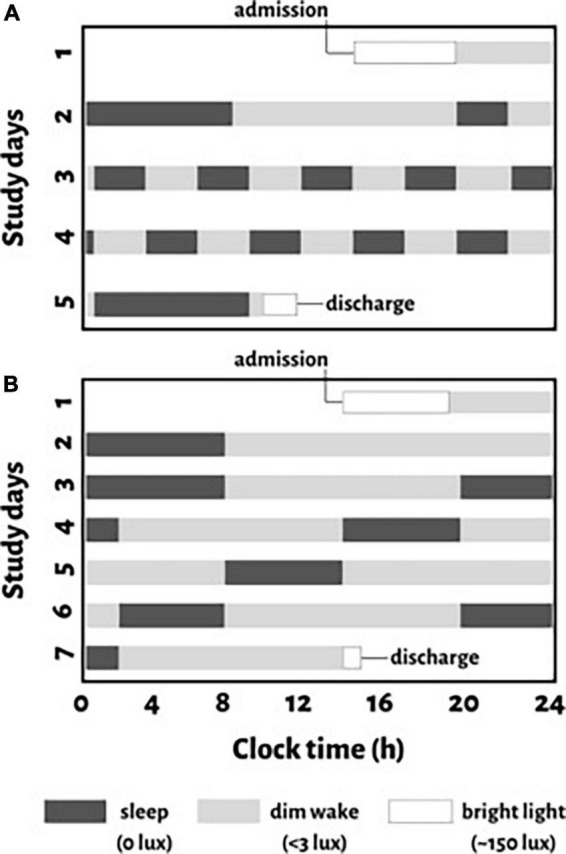
Schematic representation of study protocols for an individual with a habitual sleep time from midnight to 8:00 a.m. (clock time is provided on the *x*-axis). For protocol 1, **(A)** a baseline night of sleep at this habitual time is followed by a baseline day of wakefulness (11-hours). After baseline assessments, the circadian protocol is introduced which evenly distributes behaviors across all circadian phases with 10 identical consecutively recurring 5-hour 20-minute cycles (2-hour and 40-minute of scheduled wakefulness and 2-hour and 40-minute of a scheduled sleep opportunity) across 5 days. For protocol 2, **(B)** two nights of sleep at the participant’s habitual sleep time with 16-hour scheduled wakefulness in between, is followed by a circadian protocol with reoccurring 18-hour “days” (12-hour wake and 6-hour sleep cycles) across 7 days. Black boxes indicate scheduled sleep opportunities in darkness (<0.1 lux), gray bars indicate scheduled wakefulness in dim light (∼3 lux), and white boxes represent regular room light used when participants are admitted or discharged from the study.

To test whether there is an endogenous circadian rhythm in the CAR, we completed a secondary analysis of data from two complementary “forced desynchrony” (FD) protocols (Figure 1) throughout which participants regularly provided saliva samples for the assessment of cortisol and melatonin, with the latter used as an internal circadian phase marker, as detailed below.

### Participants

We studied adults (characterized in Table 1) in two different FD protocols (*n* = 17 and *n* = 18 respectively); one female participated in both. Participants were healthy except for mild untreated hypertension (Wetherell et al., 2018) (*n* = 9; eight from protocol 1). Participants were taking no medications with the exception of contraceptives in some females. Studies were approved by the institutional review board for human subject protection at Oregon Health and Science University, and all participants provided written informed consent. These data were collected as part of two larger studies on the effects of the circadian system, sleep, and activity on cardiovascular function in humans, of which a number of other measurements taken in protocol 1 (Figure 1A) have been reported (Thosar et al., 2018b,2019a,b; Emens et al., 2020).

**TABLE 1:** Participant characteristics.

	Protocol 1 (*n* = 17)	Protocol 2 (*n* = 18)	*P*-value
Mean (SD) age, years	53.7 ± 8.3	40.5 ± 9.7	** < 0.001**
% Female	47%^a^	72%^a^	0.129
**Baseline measurements**			
Mean (SD) BMI, kg/m^2^	29.1 ± 5.9	25.5 ± 5.3	0.066
SBP, mmHg	125.8 ± 11.9	112.4 ± 11.5	0.399
DBP, mmHg	69.4 ± 9.6	67.1 ± 9.3	0.875
Mean AHI, events/h	7.0 ± 4.1	3.0 ± 2.4	**0.001**
Mean DLMO, hh:mm	20:20 ± 1:08	19:47 ± 0:44	0.090
Mean habitual wake time, hh:mm	6:36 ± 0:56	6:36 ± 1:01	0.999
**Forced desynchrony measurements^b^**			
Mean (SD) total sleep time, min	93.0 ± 38.9	263.1 ± 71.4	
Mean (SD) sleep efficiency,%	59.0 ± 24.7	73.4 ± 20.0	
Mean (SD) time awake before lights on, min	17.0 ± 27.9	26.4 ± 57.3	
Mean (SD)% NREM	84.3 ± 15.7	79.4 ± 11.0	
Mean (SD)% REM	13.9 ± 11.1	19.6 ± 8.2	
Arousal index (SD), arousals/h	18.9 ± 13.5	14.7 ± 8.5	

SD, standard deviation; SBP, systolic blood pressure; DBP, diastolic blood pressure; AHI, apnea-hypopnea index; DLMO, dim light melatonin onset. ^a^Individual level detail for post-menopausal status, use of birth control, or phase of menstrual cycle during study participation can be found in the supplemental tables. ^b^Because the sleep opportunities were different in the two protocols, sleep measures were not compared between the two. Sleep measurements across the forced desynchrony in protocol 1 were only available for 12 of the 17 participants. Bold *p*-values indicate significant differences between the two protocols.

### Protocols

In order to ensure the stability of circadian rhythms prior to admission to the in-laboratory study, participants maintained a constant self-selected nightly 8-hour sleep schedule. This schedule was verified by actigraphy (wGT3X-BT, ActiGraph) with an accompanying written sleep diary and time-stamped voice messages at bedtime and upon-awakening for at least 1 week before entering the laboratory. Additionally, participants refrained from medications, including food supplements, caffeine, alcohol, and intense physical activity; a urine drug-screening test was performed to ensure adherence to pre-admission instructions. A pregnancy test was also administered in pre-menopausal women.

In the FD protocols (Figure 1), environmental factors were controlled and behaviors that can affect either cortisol or the circadian pacemaker (e.g., activity, posture, meals, sleep, room temperature, light) were evenly distributed across the circadian cycle, while all measurements, including saliva collections for cortisol, were made across the whole circadian cycle. To avoid resetting the phase of the circadian system, all laboratory protocols scheduled during wakefulness were performed in dim light (<3 lux) and lights were <0.1 lux during scheduled sleep opportunities (Zeitzer et al., 2000). Protocol 1 (Figure 1A) was a 5-day protocol with 10 identical consecutive 5-hour 20-minute “days” with 2-hour and 40-minutes of both scheduled wakefulness and sleep opportunity. Protocol 2 (Figure 1B) was a 7-day protocol with five identical consecutive 18-hour “days” with 12-hour scheduled wakefulness and a 6-hour sleep opportunity. In protocol 1, the at-home routine was followed by a baseline night and day in the laboratory (8-hour scheduled sleep, 11-hour scheduled wakefulness, Figure 1A). Protocol 2 provided an additional night for laboratory acclimation followed by a baseline day and night in the laboratory (8-hour scheduled sleep, 16-hour scheduled wakefulness, Figure 1B). Protocol 2 also provided 1 additional 12-hour wake period before the five reoccurring 18-hour “days.”

### Measurements

Saliva was regularly collected using cotton swabs (Salivette^®^, Starstedt, Inc, Newton, NC, USA). Following saturation, the swabs were spun and the collected saliva samples were frozen at −80°C until analyses. Salivary melatonin was measured using a radioimmunoassay from Bühlmann Laboratories (Schönenbuch, Switzerland), employing the Kennaway G-280 anti-melatonin antibody. The inter-assay coefficient of variation (CV) was 11% and the mean reported intra-assay CV was 7.9%. Dim-light melatonin onset (DLMO) was used as the circadian phase marker for the assignment of circadian phases to the other variables including the CAR. From the time series of salivary melatonin concentrations for each day, we determined the DLMO as the linear interpolated time point when melatonin exceeded 3 pg/ml (Benloucif et al., 2008). In one participant whose salivary melatonin never dipped below 3 pg/ml, 4 pg/ml was used as the threshold. Circadian period was calculated from the slope of daily DLMO assessments captured across the FD protocol as previously described (Thosar et al., 2019a,b). For four participants, only one DLMO was detected as they withdrew from the study on day four or five, so circadian period could not be measured. In these participants, we used this study’s average circadian period (τ = 24.03 h) to prevent excluding their data from analysis.

Salivary cortisol concentration, which reflects the concentration of biologically active unbound cortisol in the serum and is unaffected by variations in cortisol binding globulin (Vining et al., 1983), was determined by a high sensitivity ELISA from Salimetrics (State College, PA, USA). The reported analytical and functional sensitivity of the assay were <0.007 and 0.018 ug/dL, respectively. The mean in-house inter-assay CV was 5.1% and the mean reported intra-assay CV was 5%. We used the saliva samples collected upon-awakening and 50-minutes post-awakening to measure the CAR. Each participant in protocol 1 and protocol 2 provided 6–10 and 1–5 CAR assessments, respectively across the study protocol. Upon-awakening samples were collected while participants were still supine at 1 min after lights-on [∼3 lux] in protocol 1, and 7 min after lights on [∼3 lux] in protocol 2. Collection was later in protocol 2 to accommodate a blood pressure assessment first. The second collection 50-minutes post-awakening was collected while seated in protocol 1 and while supine in protocol 2. In between the two saliva collections, participants were provided the opportunity to use the bathroom and may have gently moved around the room. Additionally, in between the saliva measurements, we assessed flow-mediated dilation (Thosar et al., 2019a) and blood pressure in both protocols. During this time in protocol 1, we also measured mood using the Profile of Mood States and Positive and Negative Affect Schedule (Emens et al., 2020), sleepiness using the Stanford Sleepiness Scale (Hoddes et al., 1973), appetite levels using a visual analogue scale, and a 3-minute psychomotor vigilance task (measurement for sustained attention). The aforementioned measures were introduced upon admission (i.e., before the first sleep opportunity) in order to acclimate participants to procedures and surveys. Saliva samples were collected before participants ate or brushed their teeth. Participants did not drink any water for at least 15 min prior to saliva collection.

Standard full polysomnography (except leg electromyogram) was acquired and scored according to American Academy of Sleep Medicine guidelines (Iber, 2007). Time awake prior to lights on, total sleep time, sleep efficiency, arousal index, and percent sleep time spent in NREM and REM were used in secondary analyses to examine the association of sleep (or time awake prior to lights on) and CAR.

### Data analysis

Each datum was assigned a circadian phase (0–359°) based on an individual’s DLMO (0°) and estimated circadian period (one full circadian period = 360°). The CAR was calculated as the value of cortisol 50 min post-awakening minus the value immediately upon-awakening. The independent effects of the circadian cycle on cortisol concentration immediately upon-awakening, 50-minutes post-awakening, and CAR were assessed by using linear mixed model cosinor analyses with the fundamental component of the circadian cycle (∼24 h) as a fixed effect and participant (to account for repeated measurements within an individual) included as a random factor. To account for any non-sinusoidal circadian effects and any gradual changes across the protocol, the model was also run with additional fixed effects of the second harmonic (∼12 h) and a linear time-into-protocol component. However, as the latter were not significant and did not improve model fit (assessed using Akaike information criterion and Bayesian information criterion), neither factor was included in the final model. Only the data collected during the reoccurring FD “days” were used for these linear mixed models. The circadian models for the CAR of each protocol are described below by the phases at which their peaks occur and by their peak-to-trough rhythm amplitudes. Secondary analyses separately added age, sex, or hypertension to the aforementioned linear mixed models. Additionally, separate linear mixed models for each sleep parameter with participant as a random factor were first assessed for their contribution on CAR. In order to estimate possible non-linear relationships with CAR, both linear and quadratic terms for sleep parameters were included in our models. Significant parameters were then added step-wise to the circadian model. Given the non-normality of cortisol, CAR, and sleep data, the data were log transformed. The models run using raw units were similar to the models generated using data transformations, thus the raw units are used for easier interpretation. All statistical analyses were performed using STATA 16.0. The Wald Z test was used to evaluate significance of individual fixed effects, and Wald Chi-Square was used to evaluate significance of the overall model. The significance was accepted at *p* < 0.05. Descriptive statistics are expressed as mean ± standard error unless otherwise specified.

## Results

Demographic details for each protocol are summarized in Table 1 and details for individual participants are provided in Supplementary Tables 1, 2. Both men and women in protocol 1 were significantly older and had a higher apnea-hypopnea index (AHI), measured during their baseline night of sleep in the laboratory, compared to participants in protocol 2. However, no participant exceeded an AHI in the range of mild sleep apnea (AASM Task Force, 1999), therefore AHI was not considered a co-variate in our analyses.

**FIGURE 2:**
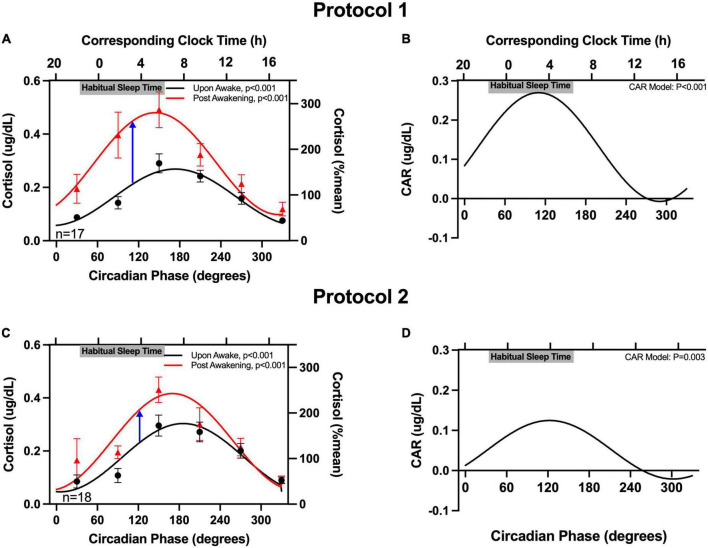
Circadian rhythm in the cortisol awakening response (CAR). The cortisol concentrations upon-awakening and 50 min post-awakening as a function of circadian time are plotted on the left column **(A,C)** and the CAR is plotted on the right **(B,D)**. Data are expressed as absolute values (left *y*-axes) and as a percentage of the mean of each participant’s upon-awake values (right *y*-axes). Participants’ binned data (60°, 4-hour intervals, means±SEM, left *y*-axes) are depicted as black circles (upon-awake) and red triangles (50 min post-awakening). Binned data were averaged within an individual first, as an individual could contribute more than one point to each bin. The solid lines represent the cosinor model fits. The blue arrows in **(A,C)** highlight the peaks of CAR in the two protocols. The corresponding clock times (determined from the average times of the DLMO for these participants) are shown on the top *x*-axes. Gray bars also on the top *x*-axes indicate the participants’ average habitual sleep times (determined from the at home assessment).

Salivary cortisol upon-awakening was similar in both protocols (averages: 0.17 μg/dL in protocol 1 and 0.18 μg/dL in protocol 2; non-significant difference). Salivary cortisol upon-awakening exhibited a robust significant circadian rhythm in both protocols, with levels rising across the circadian phases that correspond to the participants’ habitual sleep times, and peaking at the circadian phase corresponding to shortly after the participants’ habitual awakening time (6:36 a.m. for both protocols) (Figure 2). Cortisol concentrations 50-minutes post-awakening exhibited a similar circadian rhythm but with values on average increased by 0.13 μg/dL in protocol 1 and 0.06 μg/dL in protocol 2. When examining the rhythm of the CAR (difference between 50-minute post-awakening and immediately upon-awakening), the magnitude of the CAR also exhibited a significant circadian rhythm with a peak at 110° for protocol 1 (corresponding to an average time of ∼3:40 a.m. in this subgroup of participants) and a peak at 122° for protocol 2 (corresponding to an average time of ∼3:45 a.m. in the other subgroup of participants). The peak-to-trough amplitude in the CAR for protocol 1 was 0.28 μg/dL. The peak-to-trough amplitude for protocol 2 was less than half of that value (0.12 μg/dL). In both protocols, the CAR resulted in a cortisol increase of ≥50% when awakening occurred ∼3 h before the time of habitual awakening (circadian phase range = 110–122°). The magnitude of the CAR decreased across the morning with no change in cortisol levels from awakening when awakening occurred in the afternoon and evening (circadian phase range = 260–350°). Individual data (phase and amplitude for the CAR) are available in Supplementary Tables 1, 2. When examining the individual circadian rhythms, in protocol 1, 60% of participants had a significant rhythm (Supplementary Table 1); and in protocol 2, 71% of the detected rhythms were significant (Supplementary Table 2). There were no significant fixed effects nor impact on the circadian variation of the CAR when age, sex, or hypertension status were separately added to the model.

**FIGURE 3:**
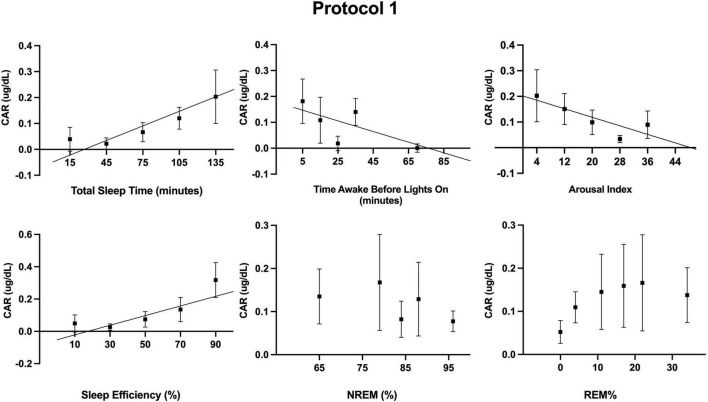
Associations between measured sleep parameters during the 10 forced desynchrony sleep opportunities of protocol 1 and the cortisol awakening response (CAR). Participants’ binned data (binned into 5–6 intervals for a given sleep parameter, means±SEM) are depicted as black squares. Binned data were averaged within an individual first, as an individual could contribute more than one point to each bin. The solid lines represent significant linear regressions as tested using mixed models (see Table 2). A quadratic term was also estimated but not significant.

**FIGURE 4:**
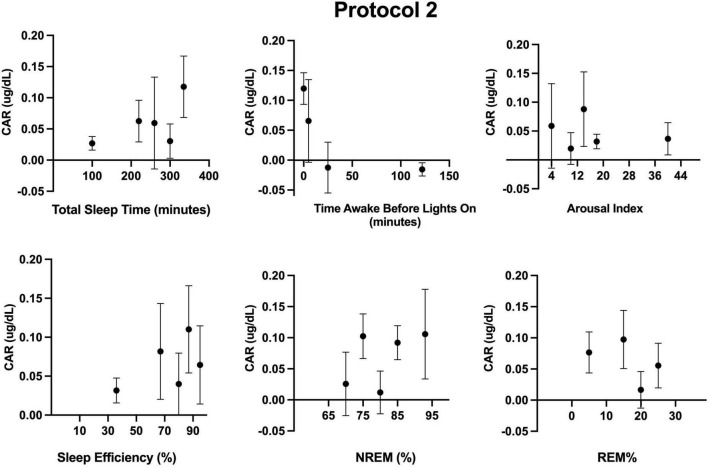
Associations between measured sleep parameters during the five forced desynchrony sleep opportunities of protocol 2 and the cortisol awakening response (CAR). Participants’ binned data (binned into 4–5 intervals for a given sleep parameter, means±SEM) are depicted as black circles. Binned data were averaged within an individual first, as an individual could contribute more than one point to each bin. Linear (see Table 2) and quadratic models were not significant.

Additionally, we examined the independent contribution of sleep (or lack thereof) on CAR. Descriptive statistics for sleep across the FD in each protocol are provided in Table 1. Sleep parameters were only available for 12 of the 17 participants of protocol 1. However, a sensitivity analysis with only these 12 participants revealed that their unadjusted CAR model was identical to the CAR model with all 17 participants. As noted in the methods, the effects of each sleep parameter on the CAR were fit using both linear and quadratic terms. The quadratic term was not significant when estimating the log transformed data and was therefore removed from the models herein. Separate mixed models (Table 2 and Figures 3, 4) for the linear relationship between the CAR and sleep parameters demonstrated that each additional minute in total sleep time was associated with an increased CAR of 0.002 ± 0.0005 μg/dL (*p* < 0.001) in protocol 1; total sleep time during each sleep opportunity was in the range of 1–155 min out of a 160 min opportunity. In protocol 2, where sleep was in the range of 52–350 min out of a 360 min opportunity, total sleep time was not significantly associated with the CAR (*p* = 0.657). Similarly, in protocol 1, each percent increase in sleep efficiency was associated with a 0.003 ± 0.0008 μg/dL (*p* < 0.001) increase in the CAR, and, in contrast, an increase in the arousal index was associated with a decrease in the CAR −0.004±0.0016 μg/dL (*p* = 0.011). However, in protocol 2, sleep efficiency and arousal index were not associated with the CAR. The percent of sleep spent in NREM and REM were not associated with CAR in either protocol. Finally, the amount of time awake before lights-on was negatively associated with CAR in protocol 1 but not protocol 2 (Protocol 1: -0.002 ± 0.0007 μg/dL per min awake, *p* < 0.01, range of time awake 0–115 min; Protocol 2: −0.0004 per min awake, *p* = 0.115, range 0–286 min). When either total sleep time or sleep efficiency were added into the circadian CAR model for protocol 1, the circadian component remained significant but the peak was shifted earlier to 85° (∼2:00 a.m.), and the peak-to-trough amplitude was reduced to 0.17 μg/dL (*p* = 0.002). Additionally, total sleep time and sleep efficiency remained a significant independent component, with each additional minute of sleep being associated with an increase in the CAR by 0.001 ± 0.0006 μg/dL (*p* = 0.015) and each percent of sleep efficiency was associated with an increase in the CAR by 0.002 ± 0.001 μg/dL (*p* = 0.016). When time awake prior to lights on was added to the circadian CAR model, the rhythm remained significant (*p* = 0.001) with a peak at 95° (∼2:40 a.m.) and a peak-to-trough amplitude of 0.19 μg/dL; time awake remained significant in the model and was associated with a decrease in CAR by 0.001 ± 0.0007 μg/dL (*p* = 0.035). Arousal index was no longer significant when added to the circadian model.

**TABLE 2:** Univariable and multivariable mixed models for the circadian and sleep effects on the cortisol awakening response (CAR) in two distinct protocols.

	Protocol 1			Protocol 2		
	β	SE	*P*	β	SE	*P*
**Univariable analyses**						
Circadian	0.129	0.0220	*P* < 0.001	0.060	0.0191	*P* = 0.002
Total sleep time	0.002	0.0005	*P* < 0.001	0.0002	0.0002	*P* = 0.437
Sleep efficiency	0.003	0.0008	*P* < 0.001	0.001	0.0001	*P* = 0.512
Time awake before lights on	–0.002	0.0007	*P* = 0.003	–0.0004	0.0003	*P* = 0.115
% NREM	–0.002	0.0014	*P* = 0.232	0.0000	0.0002	*P* = 0.916
% REM	0.003	0.0019	*P* = 0.101	–0.001	0.002	*P* = 0.530
Arousal index	–0.004	0.0016	*P* = 0.011	–0.001	0.002	*P* = 0.582
**Multivariable analyses**						
**Circadian + total sleep time**						
Circadian	0.075	0.0268	*P* = 0.005	0.058	0.020	*P* = 0.004
Total sleep time	0.002	0.0006	*P* = 0.005	0.0000	0.0002	*P* = 0.872
**Circadian + sleep efficiency**						
Circadian	0.076	0.0267	*P* = 0.005	0.058	0.020	*P* = 0.004
Sleep efficiency	0.003	0.0009	*P* = 0.006	–0.0001	0.001	*P* = 0.886
**Circadian + time awake before lights on**						
Circadian	0.086	0.0264	*P* = 0.001	0.055	0.020	*P* = 0.007
Time awake before lights on	–0.002	0.0007	*P* = 0.031	–0.0002	0.0003	*P* = 0.482
**Circadian + arousal index**						
Circadian	0.086	0.0270	*P* = 0.001	0.058	0.020	*P* = 0.004
Arousal index	–0.003	0.0016	*P* = 0.101	0.0002	0.002	*P* = 0.931

## Discussion

We found that the endogenous circadian system modulates the magnitude of the CAR with the largest CAR following awakenings at circadian phases that occurred ∼3 h prior to the participants’ habitual awakening times. This finding is robust and generalizable as it was confirmed using two independent circadian protocols with largely distinct populations.

The initiation of the CAR requires the transition from sleep to a conscious state (Wilhelm et al., 2007; Vargas and Lopez-Duran, 2020). Although the CAR is most frequently assessed following nighttime sleep when cortisol levels are on the rise, the CAR is generally considered a distinct response from the natural basal circadian levels of cortisol (Wilhelm et al., 2007). The sudden rise in cortisol in response to awakening may reflect the rapid switch of cortical and sub-cortical brain regions during the transition to awakening (Clow et al., 2010). This switch is associated with an increase in adrenal sensitivity to adrenocorticotropic hormone (ACTH). However, adrenal sensitivity can also be driven by the suprachiasmatic nucleus (SCN) in the hypothalamus (Buijs et al., 2003). Research in rodents suggests that this sensitivity is mediated by direct sympathetic innervation of the adrenal gland by the splanchnic nerve (Buijs et al., 1997; Ulrich-Lai et al., 2006; Bornstein et al., 2008). In addition to this extra-pituitary regulation of cortisol secretion, the SCN regulates basal levels of cortisol *via* input into the paraventricular nucleus of the hypothalamus which activates ACTH in the pituitary *via* corticotropin releasing hormone (Buijs et al., 2003; Dickmeis, 2009). With respect to the CAR, it has been conceptualized that extra-pituitary neural pathways such as the splanchnic nerve are essential both for the fine-tuning of ACTH sensitivity immediately prior to awakening from nocturnal sleep and for the increased sensitivity post-awakening in response to light during this period (Buijs et al., 2003). A handful of human studies support the light sensitivity hypothesis by demonstrating a positive association between the CAR magnitude and light exposure (Scheer and Buijs, 1999; Thorn et al., 2004; Petrowski et al., 2019). While light may impact magnitude of the CAR, the CAR does persist in the absence of light (Scheer and Buijs, 1999).

Our findings are consistent with previous reports that an earlier morning awakening is associated with a higher CAR (Edwards et al., 2001; Kudielka and Kirschbaum, 2003; Federenko et al., 2004; Devine and Wolf, 2016). Among these studies, Kudielka and Kirschbaum (Kudielka and Kirschbaum, 2003) concluded that an increase in cortisol earlier in the morning was incongruent with the aforementioned premise that cortisol levels increase in response to light (as cortisol levels ought to be increased later in the morning when light is brighter) (Leproult et al., 2001). Thus, the authors noted that the CAR is not simply a response to light. Robust cortisol responses to awakening were also present in our study where light levels upon-awakening were dim. Thus, while light levels may contribute to the magnitude of the CAR, we demonstrate that the endogenous circadian system also robustly modulates the CAR and is responsible for the larger CAR at earlier than normal awakening times.

The association between total sleep time and efficiency in the 5-hour 20-minute “day”, with a 2-hour 40-minute sleep opportunity, but not the 18-hour “day” with a 6-hour sleep opportunity, highlights that prior sleep, or potentially sleep pressure (in accordance with the two process model of sleep regulation (Borbely et al., 2016)) which is shaped by sleep/wake ratios, can affect the CAR. Outside the laboratory, the typical sleep/wake ratio is approximately 1:2 (i.e., 8 h asleep, 16 h awake, per 24-hour day). Due to concerns that sleep onset could be difficult at certain circadian phases in protocol 1 with brief sleep opportunities (2-hour 40 min), we increased the sleep/wake ratio such that sleep opportunity encompassed 50% of the time in the protocol (as opposed to 33.3% sleep opportunity in protocol 2, and when at home). Thus, in protocol 1, some participants may have had reduced sleep pressure and this may have contributed to the observation that some participants achieved little sleep at times. Interestingly, in protocol 1, total sleep time and efficiency were associated with CAR levels. In protocol 2, where sleep opportunities were longer (6-hours) and participants slept at least 50 min, neither total sleep time nor efficiency were associated with the CAR. Nonetheless, after adjusting for total sleep time or efficiency in the circadian models, the circadian rhythm of the CAR was nearly identical in the two protocols.

As with our two protocols, total sleep time and quality have inconsistently been associated with the magnitude of the CAR (Eek et al., 2012; Elder et al., 2014; Castro-Diehl et al., 2015), once more potentially a reflection of prior sleep pressure or the timing of this measure. From our findings, we cannot definitively deduce the minimum amount of prior sleep needed for a CAR. However, Devine and Wolf assessed the CAR following morning (8:30 am) and afternoon (∼1:30 pm) naps and found that a 90-minute nap at either time induced a CAR (Devine and Wolf, 2016). A 50-minute afternoon nap, on the other hand, did not induce a CAR. The authors did not examine a 50-minute morning nap. Federenko et al. similarly showed that an hour-long nap during the early evening did not induce a CAR (Federenko et al., 2004), but again, this nap duration was not compared to an earlier morning timepoint. Additional research is required to determine if the CAR is dependent on total sleep time or efficiency (or the amount of prior wakefulness) or if the lack of a CAR during the afternoon is driven by the circadian system. In line with the latter hypothesis, our circadian model developed with both protocols shows that the CAR is not induced during the afternoon, or that the measure 50-minutes following awakening may even be lower than the level of baseline at that circadian time.

Our findings have important implications for the assessment of the CAR as a diagnostic tool. As noted above, the CAR has been extensively studied as a potential biomarker for physical and psychological health outcomes such as chronic stress, depression, and hypertension (Chida and Steptoe, 2009; O’Connor et al., 2009; Juster et al., 2011; Lovell et al., 2011; Saban et al., 2012; Duan et al., 2013). However, CARs have also been noted to be inconsistent not only between people, but within an individual (Almeida et al., 2009; Stalder et al., 2016). In their review, Stalder et al. (2016) outline methods and a number of factors to consider in an effort to minimize variability between CAR measures. Based on our findings, the CAR can drastically change based on the phase of the endogenous circadian cycle at which measures are collected. This circadian rhythm was not significantly altered when we adjusted our models for age and sex, two trait co-variates that have been identified to impact the CAR but with mixed findings (Wust et al., 2000; Kudielka and Kirschbaum, 2003). Thus, to more accurately use the CAR as a biomarker it may be necessary to account for an individual’s internal circadian phase using a validated method, as recently reviewed by Reid (2019). In our participants, who habitually slept at night (prior to in-laboratory assessment), we found that the largest CAR resulted when awakening occurred at the circadian phase that corresponded to ∼3 h prior to the time of habitual wakening, whereas there was virtually no CAR when awakening occurred in the afternoon nor in the evening. It therefore seems highly likely that night shift workers who sleep during the daytime, and whose internal circadian rhythms are not completely reset to synchronize with their altered sleep/wake timing (Saksvik et al., 2011) will have a reduced CAR following daytime sleep, which could result in sluggish, diminished, and therefore potentially sub-optimal physiological respronses to stressors. It would be useful to test these dynamics in shift workers in the field as there could be many aspects of physiology, mood and clinical syndromes that are affected by such a hypothesized blunted CAR.

This study has many strengths including the validation of our findings using two different circadian protocols with largely distinct study populations. Moreover, we used polysomnography to assess and statistically adjust for sleep parameters. To that extent, as we recognize the importance of collecting samples soon after awakening, we were able to adjust our models for the time awake before lights on and found that it had only a modest effect compared to the effects of the intrinsic clock. We were also able to control behaviors including the amount of physical activity between the two saliva collections. Our second saliva collection time point, ∼50 min post-awakening, is slightly later than the more commonly assessed 30–45 min post-awakening (Pruessner et al., 1997; Clow et al., 2004). However, cortisol concentrations do not greatly vary between 30–60 min (Pruessner et al., 1997). Thus, our time points should provide a good estimate of the CAR. There are also limitations to this study. For example, while we estimated possible linear and quadratic relationships between the CAR and sleep there are many physiological processes that could lead to other linear relationships that we did not explore. To this extent, additional nuances in sleep such as slow wave activity, a marker for homeostatic sleep drive, as detected by the electroencephalographic power spectrum, were not analyzed in this study but should be considered in future research (Achermann et al., 1993). The present study clearly demonstrates that the CAR is regulated by the circadian system. Future studies should additionally assess ACTH to determine if the magnitude of the CAR is dissociated from ACTH.

## Data availability statement

The raw data supporting the conclusions of this article will be made available by the authors, without undue reservation.

## Ethics statement

The studies involving human participants were reviewed and approved by OHSU Institutional Review Board (IRB). The patients/participants provided their written informed consent to participate in this study.

## Author contributions

NB, SS, ST, and MB designed the initial studies. NB and SR contributed to the analysis of the data. NB drafted the manuscript. All authors contributed to monitored, or managed data collection, and approved the manuscript.
