# Supplementary material for: The circadian system modulates the cortisol awakening response in humans
Source: Front Neurosci. 2022 Nov 3;16:995452. doi: 10.3389/fnins.2022.995452 (PMC9669756; doi:10.3389/fnins.2022.995452)
Supplement: Supplementary file 1 [file Data_Sheet_1.PDF]

Supplemental Table 1 Summary of individual covariates and circadian cortisol awakening response (CAR) characteristics for protocol 1.

Participant	Age	Sex	Menopausal status	Birth control	Phase of menstrual cycle	Body Mass Index	Apnea-hypopnea index	Systolic Blood Pressure	Diastolic Blood Pressure	Circadian CAR phase	Circadian CAR amplitude
1	46	F	Perimenopausal			40	12.4	124	59	140^{a}	0.17^{a}
2	56	F	Perimenopausal			33	12.1	140	77	**164^{***}**	**0.34^{***}**
3	54	M				26	3.1	140	70	**93^{**}**	**0.37^{**}**
4	46	F	Perimenopausal			31	9.3	109	65	94	0.25
5	43	F	Premenopausal	IUD		23	1.1	103	63	**99^{**}**	**1.02^{**}**
6	54	F	Postmenopausal			25	2.9	114	61	112	0.03
7	63	M				24	10.5	124	61	**116^{***}**	**0.11^{***}**
8	46	F	*NovaSure* endometrial ablation			23	2.6	108	56	119	0.12
9	57	M				40	4.8	129	66	77	0.10
10	52	M				25	N/A	127	75	**90^{***}**	**0.36^{***}**
11	64	F	ovariectomy			31	11.2	132	77	**54^{**}**	**0.31^{**}**
12	48	F	hysterectomy			39	8.7	126	63	**150^{***}**	**0.27^{***}**
13	41	M				27	2.4	144	87	**107^{*}**	**0.22^{*}**
14	64	M				26	5.9	139	72	**106^{***}**	**0.81^{***}**
15	62	M				25	7.5	130	65	**107^{***}**	**0.47^{***}**
16	48	M				32	13.6	131	87	332	0.14
17	68	M				25	4.6	133	77	185	0.29

F=female, M=male. Significant rhythms are bolded. *p<0.05, **p<0.01, ***p<0.001 ^{a}p<0.10

Supplemental Table 2 Summary of individual covariates and circadian cortisol awakening response (CAR) characteristics for protocol 2.

Participant	Age	Sex	Menopausal status	Birth control	Phase of menstrual cycle when studied	Body Mass Index	Apnea-hypopnea index	Systolic Blood Pressure	Diastolic Blood Pressure	Circadian CAR phase	Circadian CAR amplitude
1	42	F	Premenopausal	None	Follicular	19.9	0.5	106	61	**35****	**0.11****
2	39	F	Premenopausal	IUD		39.9	3.5	126	75	**101*****	**0.38*****
3	59	F	Postmenopausal			25.8	2.3	101	65	**190****	**0.35****
4	35	M				24.5	4.4	107	62	**135****	**0.57****
5	36	F	Premenopausal	None	Luteal	24.5	3.4	119	65	undetermined	undetermined
6	34	F	Premenopausal	IUD		20.5	2.1	99	53	undetermined	undetermined
7	37	F	Premenopausal	None	Follicular	19.9	2.5	127	71	121	0.06
8	33	M				23.7	5.9	136	90	170	0.08
9	38	M				25.5	6.1	113	75	**48*****	**0.28*****
10	58	F	Perimenopausal			25.8	1.1	118	59	**116*****	**0.08*****
11	31	M				19.7	0.3	120	73	**122*****	**0.30*****
12	32	F	Premenopausal	IUD		22.7	0.6	93	53	**16***	**0.19***
13	30	M				28.6	0.0	121	72	undetermined	undetermined
14	54	F	Premenopausal	None	Follicular	22.3	2.3	107	76	329	0.22
15	36	F	Premenopausal	None	Luteal	27.9	8.9	114	60	168^{a}	0.27^{a}
16	42	F	Premenopausal	None	Follicular	23.1	5.2	108	66	**145*****	**0.32*****
17	36	F	Premenopausal	NuvaRing		31.2	2.0	112	72	undetermined	undetermined
18	57	F	Perimenopausal			33.6	2.8	97	59	**174*****	**0.24*****

F=female, M=male; “undetermined”= there were not sufficient values to determine the individual rhythm for the 4 participants that withdrew from the study on days 4 or 5. Significant rhythms are bolded. *$p<0.05$, **$p<0.01$, ***$p<0.001$ $^{a}p<0.10$
